# Recurrence pattern and TP53 mutation in upper urinary tract urothelial carcinoma

**DOI:** 10.18632/oncotarget.9904

**Published:** 2016-06-07

**Authors:** Chung-Hsin Chen, Kathleen G. Dickman, Chao-Yuan Huang, Chia-Tung Shun, Huai-Ching Tai, Kuo-How Huang, Shuo-Meng Wang, Yuan-Ju Lee, Arthur P. Grollman, Yeong-Shiau Pu

**Affiliations:** ^1^ Department of Urology, National Taiwan University Hospital, Taipei, Taiwan; ^2^ Department of Pharmacological Sciences, Stony Brook University, Stony Brook, NY, USA; ^3^ Department of Medicine, Stony Brook University, Stony Brook, NY, USA; ^4^ Department of Forensic Medicine and Pathology, National Taiwan University Hospital, Taipei, Taiwan

**Keywords:** aristolochic acid, cigarette smoking, herbs, renal pelvis, ureter

## Abstract

*TP53* mutation patterns are associated with prognosis of various cancers. This study was designed to investigate the association between *TP53* mutation patterns and recurrence patterns in upper urinary tract urothelial carcinoma (UTUC) patients. A total of 165 consecutive UTUC patients who underwent nephroureterectomies were enrolled for measuring mutation patterns of *TP53* gene from exome 2 to 11. Bladder recurrence, contralateral UTUC recurrence, and metastases were compared among groups by using log-rank test and Cox proportional hazard model. Single base substitution as an A:T to T:A transversion was noted in 55 (33.3%) patients (AT group). Forty-two (25.5%) patients had *TP53* mutations with only other than A:T to T:A transversion (NAT group), and 68 patients (41.2%) had wide-type *TP53* (WT group). AT group was predominately female (64%, 52%, 29%, respectively), had a higher incidence of end-stage renal disease (24%, 14%, 10%, respectively), and had more high-grade tumors (82%, 74%, 62%, respectively) compared to NAT and WT groups. With adjustment of tumor grade/stages, bladder and contralateral UTUC recurrence-free survival duration was shortest in NAT (*p* < 0.001) and AT group (*p* < 0.001), respectively. NAT group had a shorter metastasis-free survival duration than the other two groups combined (*p* = 0.018). As a result, A:T to T:A transversion increased contralateral UTUC recurrence risk, but other mutations in *TP53* raised the hazard of bladder recurrence and metastases. Therefore, *TP53* mutation pattern may be a useful biomarker to predict recurrence patterns of UTUC patients.

## INTRODUCTION

*TP53* is the most frequent gene involved in human cancers and has been widely evaluated in urothelial carcinoma (UC). The spectrum of *TP53* mutations can be used as prognostic molecular biomarkers for various cancers [1–3]. Abnormal p53 protein translated by the mutant gene has a longer half-life than the wild-type protein, resulting in cellular accumulation of abnormal p53 [4]. Based on this characteristic, immunohistochemical (IHC) staining for p53 has been widely utilized as a surrogate marker for the mutant *TP53* gene [5]. A meta-analysis reviewed 7 small UTUC series and concluded that p53 overexpression as determined by IHC staining could be a biomarker for predicting poor disease outcomes [6].

Recently, A > T transversions in the *TP53* gene of UTUC tumors have been documented as being associated with aristolochic acid (AA) exposure [7]. We have previously shown that AA-induced UTUC, defined by A:T to T:A transversion in tumors and aristolactam-DNA adducts in the renal cortex, has characteristic outcomes, including likely contralateral upper urinary tract recurrence [8]. In addition, *TP53* gene mutation patterns were shown to be associated with different prognoses in other cancers, such as colorectal [9] and breast cancers [10]. Together with the above evidence, *TP53* mutation pattern may be correlated with disease outcomes and recurrence patterns of UTUC.

However, studies that describe the predictive value of *TP53* mutations and patterns in the prognosis of UTUCs have not been reported until now. Herein, we analyzed *TP53* mutation patterns in UTUC and evaluated the association between patterns of mutations and recurrences. In addition, the association of p53 overexpression and *TP53* mutation pattern was also evaluated.

## RESULTS

### Patient demographics and clinical features (Table 1)

**Table 1:** Demographics of the patients stratified by *TP53* mutation patterns

*TP53* mutation pattern	Mutations with A > T (AT group)	Mutations other than A > T (NAT group)	Wild type (WT group)	*p* value
Patient number (*n*)	55	42	68	
Median age (range)	63.5 (30–87)	67.9 (5–88)	66.4 (42–90)	0.168
Gender				0.001
Male	20 (36%)	20 (48%)	48 (71%)	
Female	35 (64%)	22 (52%)	20 (29%)	
Smoking history				0.055
Yes	6 (11%)	11 (26%)	19 (28%)	
No	49 (89%)	31 (74%)	49 (72%)	
Heavy smoking^a^				0.009
Yes	3 (5%)	8 (19%)	18 (26%)	
No	52 (95%)	34 (81%)	50 (74%)	
CKD stage				0.361
Normal	16 (29%)	16 (38%)	26 (38%)	
3	21 (38%)	15 (36%)	29 (43%)	
4	3 (5%)	4 (10%)	6 (9%)	
5	15 (27%)	7 (17%)	7 (10%)	
ESRD				0.124
Yes	13 (24%)	6 (14%)	7 (10%)	
No	42 (76%)	36 (86%)	61 (90%)	
DM				0.07
Yes	11 (20%)	6 (15%)	22 (33%)	
No	44 (80%)	35 (85%)	45 (67%)	
Hypertension				0.15
Yes	18 (33%)	21 (51%)	31 (46%)	
No	37 (67%)	20 (49%)	36 (54%)	
AL-DNA adduct				<0.001
Yes	47 (89%)	22 (54%)	35 (51%)	
No	6 (11%)	19 (46%)	33 (49%)	
Tumor location				
Renal pelvis	36 (65%)	22 (52%)	50 (74%)	0.077
Upper ureter	15 (27%)	8 (19%)	10 (15%)	0.219
Lower ureter	17 (31%)	20 (48%)	22 (32%)	0.176
Synchronous bladder tumor	14 (25%)	9 (21%)	18 (26%)	0.831
Multiple tumor location	18 (33%)	11 (26%)	22 (32%)	0.745
Grade				0.046
High	45 (82%)	31 (74%)	42 (62%)	
Low	10 (18%)	11 (26%)	26 (38%)	
Stage				0.16
Ta-1NoMo	25 (44%)	19 (45%)	40 (59%)	
T2-4NoMo	25 (45%)	22 (52%)	25 (37%)	
nodal or metastatic	6 (11%)	1 (2%)	3 (4%)	
p53 IHC staining				0.011
Negative	15 (38%)	10 (29%)	28 (54%)	
< = 50%	13 (33%)	12 (34%)	20 (38%)	
> 50%	12 (30%)	13 (37%)	4 (8%)	
Missing	15	7	16	
Follow-up (months, range)	55 (4–208)	61 (4–157)	57 (4–169)	0.605

CKD = chronic kidney disease; ESRD = end-stage renal disease; DM = diabetes mellitus; AL-DNA = aristolactam-DNA; IHC = immunohistochemical.

a heavy smoking was defined as more than 20 pack-years.

**Figure 1:**
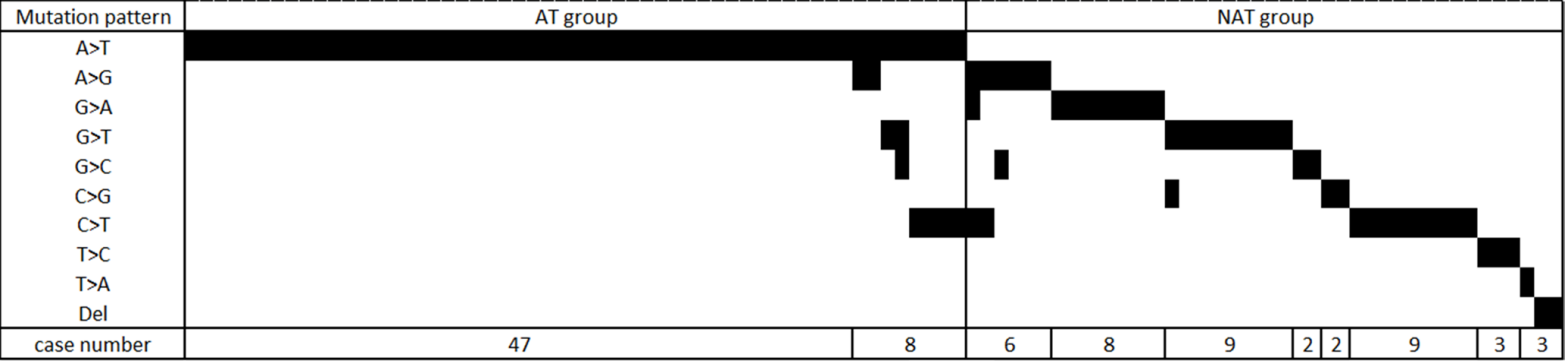
The distribution of TP53 mutation pattern in AT group and NAT group patients AT group refers to those with A:T > T:A transversion in *TP53* gene. NAT group refers to the patients have *TP53* mutations, but no A:T > T:A transversion.

In this cohort, 55 (33.3%), 42 (25.5%), and 68 (41.2%) patients were classified as AT group (*TP53* mutation with A:T to T:A SBS), NAT group (*TP53* mutation with only other than A:T to T:A SBS), and WT group (wild-type *TP53*), respectively. The distribution of *TP53* mutation patterns was shown in Figure 1 according to patient groups. Eight (14.5%) patients in AT group had both A:T to T:A SBS and non-A:T to T:A SBS. There were more females in AT group than in NAT group or WT group. A total of 35 (64%) AT group patients were female; In contrast, 71% of WT group patients were males. Only 36 (21.8%) patients in this cohort had a history of smoking, and the smoking rate was insignificantly lower in AT group than WT group (*p* = 0.055). WT group (26%) had more smoking histories of ≥ 20 pack-years than AT group (5%) (*p* = 0.009). In addition, *TP53* mutation rate was higher in the patients without smoking histories (72%) than those with smoking histories (47%) (Table S1). In the patients with *TP53* mutations, A:T to T:A transversion was more frequently noted in non-smokers, but mutation only other than A:T to T:A transversion was more identified in smokers. End-stage renal disease (ESRD) was more common in AT group (24%) than in NAT group and WT group combined (12%) (*p* = 0.0495). Diabetes mellitus was more frequently noted in WT group (33%) than the other two groups combined (18%) (*p* = 0.026). The median follow-up duration from the date of nephroureterectomies was 59 months (from 4 to 208 months) without significant difference among groups.

### Tumor characteristics (Table 1)

An increased number of high-grade tumors were observed in AT group (89%) compared to NAT group (74%) or WT group (62%). AT group had more nodal or metastatic diseases (11%) upon diagnosis of UTUC than NAT group (2%) or WT group (4%), although the rate was not statistically significant. Upon the UTUC diagnosis, 51 (31%) of all patients had tumors at ≥ 2 locations, including renal pelvis, upper ureter, lower ureter, and bladder. Multiple tumor locations and synchronous bladder tumor rates in the three groups were similar.

### Association among *TP53* mutation patterns, types and p53 immunohistochemical staining (Tables 1 and 2)

**Table 2:** Association between *TP*53 mutation type, pattern and p53 immunohistochemical staining

	Mutation type			
	Missense	Nonsense	Silent	Wild type
p53 IHC staining				
> 50%	23 (79%)	0	2 (7%)	4 (14%)
< = 50%	11 (24%)	7 (16%)	7 (16%)	20 (44%)
Negative	8 (15%)	12 (23%)	5 (9%)	28 (53%)
*TP*53 mutation pattern				
Mutations with A > T	18 (45%)	10 (25%)	12 (30%)	0
Mutations other than A > T	24 (69%)	9 (26%)	2 (6%)	0
Wild type	0	0	0	52 (100%)

IHC = immunohistochemical.

Positively-stained cells were more frequently observed in AT group and NAT group than WT group (62%, 71%, and 46%, respectively). Only 8% of WT group tumors had more than 50% of p53 IHC staining (30% in AT group and 27% in NAT group). Accordingly, *TP53* mutations were associated with higher percentage of p53 IHC staining in tumors. However, no significant difference of p53 IHC staining result was noted between AT group and NAT group.

A significant association was identified among *TP53* mutation types and p53 IHC staining (Table 2). Tumors with more than 50% of p53 IHC staining had a higher proportion (79%) of missense mutations than did those with < = 50% and negative results of IHC staining (24% and 15%, respectively; *p* < 0.001). Missense mutations were most frequently identified in UTUC tumors of AT (45%) and NAT (69%) groups.

### Bladder tumor recurrence

**Figure 2:**
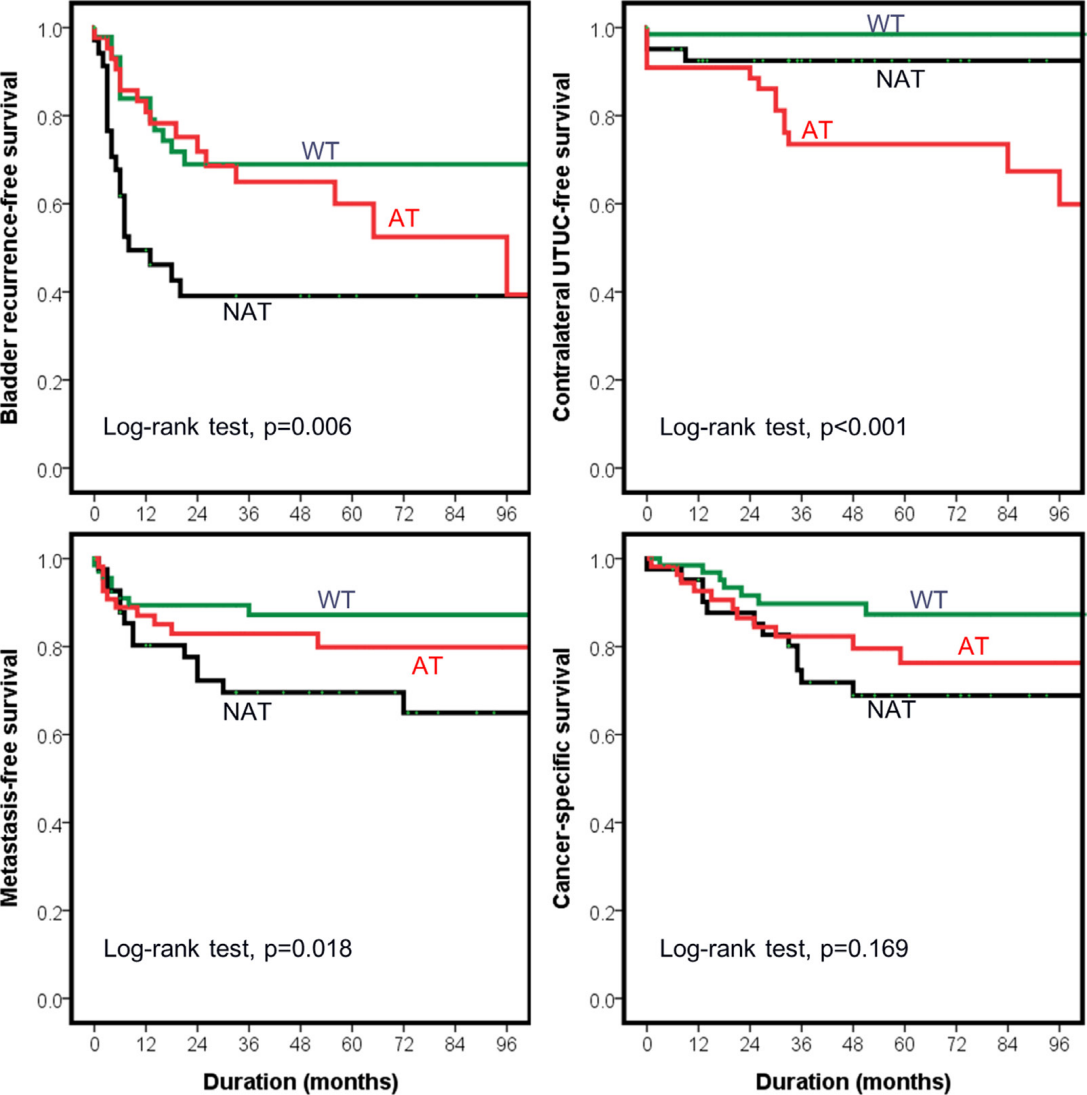
Outcomes of upper urinary tract urothelial carcinoma (UTUC) patients stratified by mutational status of *TP53* AT group refers to those with A:T > T:A transversion in *TP53* gene. NAT group refers to the patients have *TP53* mutations, but no A:T > T:A transversion. WT group represents those without *TP53* mutation.

Of all patients, 82 (49.7%) had bladder recurrences during a median follow-up of 59 months (range: 4 to 208 months). Forty-four (26.7%) with previous or synchronous bladder tumors (*n* = 41) or prophylactic cystectomy (*n* = 3) were excluded from bladder recurrence analysis. Thirty-nine (32.2%) of the remaining patients (*n* = 121) had newly diagnosed bladder cancer recurrences. The median time to bladder recurrence was only 7 months (range: 3 to 65) after nephroureterectomy. Compared to AT group and WT group, patients in NAT group had shorter bladder recurrence-free survival duration (log-rank test, *p* = 0.037 and 0.005, respectively) (Figure 2A). In univariable analysis of Cox proportional hazard model (Table 3), diabetes and *TP53* mutation pattern were the only two significant prognosticators of bladder recurrence. Lower ureteral tumor location was a statistically insignificant factor for bladder recurrence (*p* = 0.065). Multivariable analysis of the full model (model 1) revealed that diabetes and *TP53* mutations other than A:T to T:A transversions (NAT group) were associated with more bladder recurrences. In the model filled with interested variables and clinically important prognosticators, including gender, tumor grade, stage, and lower ureteral tumors, *TP53* mutations other than A:T to T:A transversions still presented as an independent variable predicting a higher risk (HR: 8.4 and 3.7, *p* < 0.001 and = 0.01, respectively) of bladder recurrence compared with wild-type *TP53* and *TP53* mutations with A:T to T:A transversions.

**Table 3:** The Cox proportional hazard model of bladder recurrence in UTUC patients

Variables	Univariable analysis						Multivariable analysis				
	Case number^a^	Recurrence events	Model 1				Model 2^b^				
			HR	range	*p* value	HR	range	*p* value	HR	range	*p* value
Age	121	39	0.98	0.95–1.01	0.13	0.96	0.92–1.00	0.076	—	—	—
Sex											
Female	57	15	1	—	—	1	—	—	1	—	—
Male	64	24	1.56	0.81–3.00	0.179	2.38	0.93–6.08	0.07	2.91	1.29–6.59	0.01
Smokers											
No	97	30	1	—	—	1	—	—	—	—	—
Yes	24	9	1.29	0.61–2.72	0.511	1.6	0.78–3.26	0.196	—	—	—
Heavy smokers^c^											
No	102	32	1	—	—	—	—	—	—	—	—
Yes	19	7	1.27	0.56–2.89	0.565	—	—	—	—	—	—
Herbs usage											
No	101	30	1	—	—	1	—	—	—	—	—
Yes	20	9	1.6	0.76–3.39	0.218	2.77	0.48–16.00	0.254	—	—	—
AL-DNA adduct											
No	42	13	1	—	—	1	—	—	—	—	—
Yes	79	26	1.04	0.52–2.08	0.907	0.88	0.33–2.31	0.794	—	—	—
TP53 mutational pattern											
Wild type	49	11	1	—	—	1	—	—	1	—	—
Mutation with A > T	39	12	1.34	0.58–3.10	0.497	3.02	0.85–10.71	0.088	2.91	0.97–8.76	0.058
Mutation other than A > T	33	16	2.94	1.33–6.49	0.008	9.93	2.87–34.37	<0.001	8.4	2.64–26.74	< 0.001
p53 IHC staining											
Negative	42	15	1	—	—	1	—	—	1	—	—
<= 50%	32	9	1.06	0.49–2.30	0.877	1.26	0.48–3.31	0.641	1.12	0.47–2.70	0.798
> 50%	17	7	2.18	0.94–5.06	0.069	3.16	0.94–10.60	0.063	1.81	0.66–4.94	0.245
ESRD											
No	107	34	1	—	—	1	—	—	—	—	—
Yes	14	5	1.09	0.43–2.80	0.854	0.43	0.05–3.42	0.427	—	—	—
DM											
No	92	25	1	—	—	1	—	—	1	—	—
Yes	29	14	2.37	1.22–4.61	0.011	6.76	2.28–20.05	0.001	4.29	1.77–10.37	0.001
Hypertension											
No	68	17	1	—	—	1	—	—	—	—	—
Yes	53	22	1.56	0.83–2.97	0.17	2.13	0.87–5.21	0.098	—	—	—
Grade											
Low	35	14	1	—	—	1	—	—	1	—	—
High	86	25	0.89	0.46–1.73	0.733	0.9	0.35–2.29	0.823	0.72	0.30–1.71	0.453
Stage											
Ta-1NoMo	62	19	1	—	—	1	—	—	1	—	—
T2-4NoMo	51	20	1.24	0.66–2.34	0.51	1.32	0.61–2.81	0.481	1.21	0.63–2.31	0.571
Multiple tumor location											
No	109	32	1	—	—	1	—	—	—	—	—
Yes	12	7	1.69	0.60–4.78	0.324	1.25	0.19–8.27	0.816	—	—	—
Lower ureter location											
No	83	23	1	—	—	1	—	—	1	—	—
Yes	38	16	1.85	0.96–3.56	0.065	1.07	0.38–3.03	0.9	1.12	0.43–2.94	0.817

UTUC = upper urinary tract urothelial carcinoma; AL-DNA = aristolactam-DNA; IHC = immunohistochemical; ESRD = end-stage renal disease; DM = diabetes mellitus.

a The patients with synchronous or previous bladder cancers or receiving radical cystectomies were excluded from this study.

b Model 2 includes variables with *p* < 0.05 in the univariable analysis, and classical prognostic factors.

c Heavy smoking was defined as more than 20 pack-years.

A total of 57% of the patients with previous or synchronous bladder cancer had *TP*53 mutations, whereas 72% of those with bladder recurrence after nephroureterectomies had *TP*53 mutations. In addition, mutation other than A:T to T:A transversion was more frequently noted in the UTUC patients with bladder recurrence after nephroureterectomies compared to those with previous or synchronous bladder cancer history (Table S2).

### Metachronous contralateral UTUC recurrence

In this cohort, previous, synchronous or metachronous bilateral UTUC diseases were identified in 18 patients, of whom 14 (78%) from AT group, 3 (17%) from NAT group, and 1 (6%) from WT group. Nine patients in AT group and 1 patient in NAT group had contralateral UTUC occurrences after nephroureterectomy. None of the WT group patients had metachronous contralateral UTUC. In the patients with synchronous or previous contralateral UTUC (*N* = 8), 5 and 2 were AT and NAT groups, respectively. Only one patient had no mutation in *TP*53 gene. Excluding those with previous and synchronous UTUC diseases, we identified AT group had a shorter contralateral UTUC-free survival duration than the other two groups (log-rank test, both *p* < 0.001) (Figure 2B). Univariable analysis revealed herbs usage, aristolactam-DNA adducts, *TP53* mutation with A:T to T:A transversions, and diabetes increased risk of metachronous contralateral UTUC recurrence. Multivariable analysis can't be performed due to the limited number of events.

### Metastasis

Of patients with non-metastatic diseases before nephroureterectomy (*n* = 155), metastases occurred in 7 (14.3%), 12 (29.3%), and 5 (7.7%) patients in AT, NAT and WT groups, respectively. Median duration between metastasis and nephroureterectomy was 8.5 months. NAT group patients had a shorter metastasis-free survival than the other two groups (Figure 2C). Tumor stage, grade, p53 IHC staining and *TP53* mutation patterns were statistically significant prognosticators of metastases. After stratifying for tumor grade and stage, NAT group predicted earlier metastasis compared to AT and WT groups combined in the multivariable model (HR = 2.50, 95% CI. 1.12–5.58, *p* = 0.026).

### Survival outcomes

A total of 44 patients died during a median follow-up of 59 months. Older age, advanced tumor grade and stage predicted shorter overall survival duration in univariable and multivariable analyses. Cancer-specific deaths were noted in 30 UTUC patients, and only tumor grade and stage were prognosticators. Patients in NAT group had an insignificantly increased risk of cancer-specific death compared to WT group (HR = 2.41, 95% CI. 0.93–6.21, *p* = 0.07). No difference in survival outcomes was noted between AT and NAT groups (Figure 2D).

## DISCUSSION

By stratifying UTUC patients according to *TP53* mutation patterns, we observed that patients with an A:T to T:A transversion in the *TP53* gene had a higher risk of contralateral upper urinary tract recurrence, and those with non- A:T to T:A transversion mutations had more bladder tumor recurrences and metastases than the other two groups. These findings demonstrate that *TP53* mutation pattern is a useful biomarker for predicting patient outcomes. Therefore, the *TP53* mutation pattern should be considered important information for clinicians in improving follow-up protocols for UTUC patients.

Previous studies discovered that p53 protein overexpression in UTUC [6, 11] was associated with recurrence- and progression-free survival duration. However, these studies used IHC staining to determine p53 expression levels. Based on the principle of this assay, positive IHC results do not recognize null mutations, such as deletions, insertions, nonsense and splicing site mutations. Approximately 30% of somatic *TP53* mutations are not identified by IHC assay, suggesting that IHC is not sufficient for detection of *TP53* mutations [12]. Hashimoto et al. reported that null mutations predicted outcomes better than the IHC assay [13]. Besides, positive IHC results from early tumor lesions should be interpreted carefully because local responses to inflammatory microenvironments can cause accumulation of wild-type p53 in tumors [14]. Hence, DNA sequencing remains the gold standard for identifying *TP53* mutations. That is why our present study investigates patients according to *TP53* sequencing results.

*TP53* mutation patterns were significantly associated with patient demographics. Tumors in smokers had fewer A:T to T:A transversions, but had more non- A:T to T:A transversion mutations. As that in lung cancer study, a G:T transversion at codon 157 in *TP53* is frequent in smokers [15, 16]. On the other hand, only 1 out of 11 patients with an A:T to T:A transversion had ever smoked in our cohort. Patients who had been exposed to herbs had a higher frequency of A:T to T:A transversions, which is the signature mutation of AA [7]. Furthermore, the A:T to T:A transversion was associated with poor renal function given that AA induces renal tubular damage [17]. In our cohort, *TP53* mutations with an A:T to T:A transversion were more frequent in female UTUC patients than males, which may be partially due the fact that more Taiwanese women tend to take herbs containing AA than men [18]. Whether gender or carcinogens contributed to differences in *TP53* mutation patterns should be further investigated.

Based on the results combining aristolactam-DNA adducts in renal cortical tissue and A:T to T:A transversions in the *TP53* gene, patients with AA-induced UTUC had more high-grade and high-stage tumors than non-AA UTUC patients [8]. This extended cohort revealed that patients with *TP53* mutations had more unfavorable tumor characteristics than those without *TP53* mutations. A:T to T:A transversion pattern was associated with the worst initial tumor presentations, such as high grade and nodal or metastatic disease, in this cohort. As the signature mutation of AA, A:T to T:A transversion suggests that AA is responsible for carcinogenesis in these cases of UTUC. Patients with this signature mutation were considered as having bilateral, either synchronous or metachronous, UTUC [8].

UTUCs with *TP53* non- A:T to T:A transversions have more and earlier bladder recurrences than those without *TP53* mutations or A:T to T:A transversions. Using IHC staining, previous studies revealed that p53 overexpression in tumors had conflicting disease-free survival results in UTUC patients [6]. In fact, p53 protein overexpression could be caused by different *TP53* mutation patterns. As shown in this study, UTUC patients with an A:T to T:A transversion did not have poorer outcomes of bladder recurrences or metastases than those without *TP53* mutations. However, non- A:T to T:A transversion mutations in the *TP53* gene did predict poorer outcomes. Hence, the precise stratification of *TP53* mutations would help predict patient outcomes.

In contrast to other tumor suppressor genes which are changed by truncating mutations, most of *TP53* gene mutations are missense substitutions (75%) [19] that are resulted from SBS clustering within DNA-binding domain of protein. Furthermore, the most frequent mutants have been revealed to have ability to cooperate with oncogenes for cellular transformation [20]. Our UTUC patients with *TP53* mutations other than A:T to T:A transversion had higher proportion of missense mutations (69%) than those with A > T transversion (45%) and no mutation (0%) (Table 2). Tumors with *TP53* mutations were supposed to be more invasive than those without mutations.

UTUC patients were under risks of bladder recurrence and contralateral recurrence after nephroureterectomy. Pathological characteristics and tumor location might help identify patients who are at risk. In this study, *TP53* mutation pattern further stratified those at risk of bladder recurrence or contralateral upper tract recurrence and facilitated decisions regarding individualized follow-up protocols. Importantly, tumor DNA sequencing is not complex, although predictive values should not be overlooked.

Compared with the current published UTUC series with results of bladder outcome, most of clinicopathological characters, such as tumor stages, multi-focality, tumor location, bladder recurrence rate excluding patients with previous bladder cancer histories were similar to our series (Table 4) [21, 22]. Therefore, the findings of our study could be further extended to other population.

**Table 4:** Comparison of clinicopathological presentation of western UTUC series and our series

Series	Countries	Case number	Ta-1 proportion	High grade	Multifocality	Renal pelvis location	Bladder recurrence^*^
Zigeuner et al.^22^	Austria	191	51.3%	50.5%	33.0%	64.4%	32.0%
Novara et al.^21^	Italy	231	46.3%	(G3) 48.5%	39.0%	64.9%	35.6%
Our large cohort^25^	Taiwan	538	46.6%	57.2%	21.0%	68.0%	30.4%
Current study	Taiwan	165	50.9%	71.5%	30.9%	65.5%	32.2%

* All the patients with previous or synchronous bladder cancer histories were excluded.

G3 = grade 3.

Although CKD and bilateral UTUC reasonably worsen patients' survival outcomes, the association between ESRD, and metachronous bladder recurrence in the patients with primary UTUC is not clear till now. In our cohort, ESRD did not increase risk of bladder recurrence, nor metastasis after nephroureterectomies, but did raise hazard of metachronous contralateral recurrence. Based on the clinical observation [8], molecular evidence [7] and epidemiological findings [23, 24], our hypothesis is that AA exposure results in both CKD and UTUC, but the sequence is uncertain. For people with more AA exposure, ESRD develops earlier than UTUC. In contrast, ESRD occurs later than or similar to UTUC in the patients with less AA exposure. Therefore, not all UTUC patients had poor renal function upon the diagnosis. For example, 4 of 8 previous or synchronous UTUC patients and 4 of 10 metachronous UTUC patients did not have CKD stage 5 disease upon the diagnosis in our cohort. Additionally, 73% of UTUC patients with signature mutation of AA did not have CKD stage 5 diseases.

A large cohort has shown that poorly controlled diabetes increased bladder recurrence in UTUC patients [25]. Although the real mechanism is still unknown, two possible mechanisms may explain what we observe in this cohort. Firstly, diabetes results in bladder dysfunction and increases risks of urinary tract infection [26], so that further promote urothelial cancer cell implantation on the injured bladder mucosa [27–29]. Secondly, upregulated insulin-like growth factor 1 (IGF-1) in diabetes patients increases tumor cell proliferation and inhibits apoptosis [30], and associated with an increased risk of bladder cancer [31].

This study has several limitations. Firstly, the sample size and number of events did not allow extensive analyses. Secondly, observations of additional contralateral recurrences and long-term survival analysis were limited by the relatively short follow-up duration (median: 59 months). Nevertheless, the events were sufficient to show that UTUC patients with *TP53* A:T to T:A transversions were at a higher risk of contralateral upper urinary tract recurrence. Thirdly, misclassification cannot be absolutely excluded because genes other than *TP53* were not sequenced. However, whole exome sequencing is costly and is not suitable as a convenient, inexpensive tool for identifying patients at risk. Fourthly, the results from the areas with profound AA exposure would render the extension of conclusion to the areas without AA exposure. Fifthly, the biology of different *TP53* mutation pattern was not investigated in this study.

## MATERIALS AND METHODS

### Patient population and enrollment criteria

A total of 232 consecutive UTUC patients who underwent nephroureterectomies and bladder cuff resection at National Taiwan University Hospital (NTUH) between August 1999 and July 2012 were included in this cohort. This study was approved by the Research Ethics Committee at NTUH and the Human Subjects Institutional Review Board at Stony Brook University. All subjects provided their written informed consent before donating samples. Patients who had lived in arsenic-endemic areas in Taiwan (*n* = 2, 0.9%) or had received chemotherapy or radiotherapy for any malignancy before tissue sampling (*n* = 4, 1.7%) were excluded from the study. None of the patients reported a history suggestive of occupational exposure to aromatic amines. A total of 61 (26.3%) patients were excluded due to inadequate tissue samples for DNA extraction. The remaining 165 cases were available for analysis.

### Specimen characteristics and storage

Tissue specimens were collected immediately after nephroureterectomies. Renal cortical samples were chosen from normal-appearing part far-away from UTUC tumors. Tumors were sampled with avoidance of inclusion of surrounding normal tissues. All fresh tissue samples were collected in aseptic vials, snap-frozen in liquid nitrogen and then stored at −80°C till DNA extraction.

### *TP53* gene sequencing

All DNA samples were prepared from frozen tumor tissues using the phenol-chloroform method. Tumor DNA *TP53* mutational status was evaluated using the Roche AmpliChip p53 microarray (Roche Molecular Systems, Pleasanton, CA, USA). This technology, described in references 11 and 12 [32, 33], is designed to identify splice site mutations and single base–pair mutations and deletions in coding regions of exons 2–11. Chiaretti and coworkers reported that the p53 AmpliChip was superior to traditional Sanger sequencing for detection of single base substitutions, the dominant mutation class in *TP53*. The *TP53* mutation data for the UTUC samples reported in the current study have been published [7, 8].

### Distribution and grouping of *TP53* SBS pattern

*TP53* SBS mutations were identified in 97 (58.8%) patients. The distribution of *TP53* SBS was shown in Figure 1. Based on our hypothesis that A:T to T:A transversion might be associated with characteristic presentation [7], we grouped the patients with A:T to T:A transversion as AT group. Patients with *TP53* mutations only other than A:T to T:A SBSs were categorized as NAT group. Patients without any *TP53* SBS (wild-type) were defined as WT group (no *TP53* mutations).

### Immunohistochemical staining

Deparaffinized five micrometer-thick sections were subjected to antigen retrieval and then incubated with anti-*TP53* antibody (clone DO-7, 1:100, Dako USA). Antigen-antibody complexes were identified by the avidin-biotin-peroxidase method (Vectastain, Vector Labs, USA) with diaminobenzidine (DAB) as the substrate. Sections were then counterstained with hematoxylin and examined by microscopy. Only staining in tumor cells was used to evaluate the proportion of *TP53* staining for semiquantitative evaluation of DAB staining in tumor cells. The percentage of cells with positive nuclear staining, based on a scale of 0–100% in increments of ten, was scored by one of the authors (KD) in a blinded manner.

### Measurement of bladder recurrence, contralateral upper tract recurrence

UTUC patients were regularly followed at our clinics per the follow-up protocol with cystoscopy examination and urine cytology at the 3rd, 6th, 12th, 18th, 24th, 30th, 36th, 48th, and 60th month after nephroureterectomy and bladder cuff resection. Imaging studies were performed every 6 months for 3 years and annually thereafter. We did not administer intravesical chemotherapy or Bacillus Calmette–Guérin instillation post-operatively if the patients did not have synchronous bladder tumors. Any suspicious symptoms or signs, such as hematuria or loin discomfort, triggered an additional survey for recurrences.

Bladder recurrence was defined as histologically-proven bladder UC that appeared after nephroureterectomy. Those with previous or synchronous bladder tumors were excluded from the analysis of bladder recurrence. Metachronous contralateral UTUC was defined as histologically-proven UC in the contralateral upper urinary tract. Similarly, the patients with synchronous bilateral or previous contralateral UTUC were not included in the final model. Bladder cancer recurrence-free and metachronous contralateral UTUC-free survival were calculated from the date of nephroureterectomy to the date of diagnosis or to the date of the last follow-up (censored).

### Other clinical data collection

Information regarding Chinese herb use, occupational exposure, and smoking behavior was obtained directly from patients using a standardized questionnaire. Estimated glomerular filtration rate was used for staging chronic kidney disease [34]. Heavy smokers were defined as those with a cumulative smoking history of 20 pack-years or more.

All surgical specimens were reviewed by a single pathologist at NTUH. Tumors were graded according to the WHO 2004 classification and staged using the TNM 2002 classification.

### Statistical methods

All statistical analyses were performed with Stata 8.2 for Windows. The Kruskal-Wallis rank sum test was used to compare the medians among patient groups. Contingency tables were constructed for comparisons using the chi-square test. The log-rank test and Cox proportional hazard model were used to compare metachronous contralateral UTUC and bladder cancer recurrence-free survival duration among the three patient groups. The factors with either statistical significance (*p* < 0.05 in univariable analysis) or clinical importance were selected for the multivariable model. All tests were two-tailed. A *p*-value of < 0.05 was considered statistically significant.

## CONCLUSIONS

Different *TP53* mutation patterns in UTUC tumors were related to different tumor outcomes. A:T to T:A transversions increased contralateral UTUC recurrence, while mutations other than A:T to T:A transversions raised bladder tumor recurrence. The *TP53* gene mutation pattern may be a useful biomarker for predicting the recurrence patterns in UTUC patients.

## SUPPLEMENTARY MATERIALS TABLES



## Abbreviations

UC: urothelial carcinoma
UTUC: upper tract urothelial carcinoma
SBS: single base substitution
IHC: immunohistochemical
AA: aristolochic acid.

## References

[1] Hollstein M, Sidransky D, Vogelstein B, Harris CC (1991). p53 mutations in human cancers. Science.

[2] Levine AJ, Momand J, Finlay CA (1991). The p53 tumour suppressor gene. Nature.

[3] Petitjean A, Achatz MI, Borresen-Dale AL, Hainaut P, Olivier M (2007). TP53 mutations in human cancers: functional selection and impact on cancer prognosis and outcomes. Oncogene.

[4] Maslon MM, Hupp TR (2010). Drug discovery and mutant p53. Trends in cell biology.

[5] Soussi T, Lozano G (2005). p53 mutation heterogeneity in cancer. Biochemical and biophysical research communications.

[6] Ku JH, Byun SS, Jeong H, Kwak C, Kim HH, Lee SE (2013). The role of p53 on survival of upper urinary tract urothelial carcinoma: a systematic review and meta-analysis. Clinical genitourinary cancer.

[7] Chen CH, Dickman KG, Moriya M, Zavadil J, Sidorenko VS, Edwards KL, Gnatenko DV, Wu L, Turesky RJ, Wu XR, Pu YS, Grollman AP (2012). Aristolochic acid-associated urothelial cancer in Taiwan. Proceedings of the National Academy of Sciences of the United States of America.

[8] Chen CH, Dickman KG, Huang CY, Moriya M, Shun CT, Tai HC, Huang KH, Wang SM, Lee YJ, Grollman AP, Pu YS (2013). Aristolochic acid-induced upper tract urothelial carcinoma in Taiwan: clinical characteristics and outcomes. International journal of cancer.

[9] Bazan V, Agnese V, Corsale S, Calo V, Valerio MR, Latteri MA, Vieni S, Grassi N, Cicero G, Dardanoni G, Tomasino RM, Colucci G, Gebbia N (2005). Specific TP53 and/or Ki-ras mutations as independent predictors of clinical outcome in sporadic colorectal adenocarcinomas: results of a 5-year Gruppo Oncologico dell'Italia Meridionale (GOIM) prospective study. Annals of oncology.

[10] Olivier M, Langerod A, Carrieri P, Bergh J, Klaar S, Eyfjord J, Theillet C, Rodriguez C, Lidereau R, Bieche I, Varley J, Bignon Y, Uhrhammer N (2006). The clinical value of somatic TP53 gene mutations in 1,794 patients with breast cancer. Clinical cancer research.

[11] Lee YC, Wu WJ, Li WM, Lin HH, Huang CN, Chai CY, Chang LL, Lin HL, Ke HL (2013). Prognostic value of p53 protein overexpression in upper tract urothelial carcinomas in Taiwan. Anticancer research.

[12] Alsner J, Jensen V, Kyndi M, Offersen BV, Vu P, Borresen-Dale AL, Overgaard J (2008). A comparison between p53 accumulation determined by immunohistochemistry and TP53 mutations as prognostic variables in tumours from breast cancer patients. Acta oncologica.

[13] Hashimoto T, Tokuchi Y, Hayashi M, Kobayashi Y, Nishida K, Hayashi S, Ishikawa Y, Tsuchiya S, Nakagawa K, Hayashi J, Tsuchiya E (1999). p53 null mutations undetected by immunohistochemical staining predict a poor outcome with early-stage non-small cell lung carcinomas. Cancer research.

[14] Hofseth LJ, Saito S, Hussain SP, Espey MG, Miranda KM, Araki Y, Jhappan C, Higashimoto Y, He P, Linke SP, Quezado MM, Zurer I, Rotter V (2003). Nitric oxide-induced cellular stress and p53 activation in chronic inflammation. Proceedings of the National Academy of Sciences of the United States of America.

[15] Hainaut P, Pfeifer GP (2001). Patterns of p53 G—>T transversions in lung cancers reflect the primary mutagenic signature of DNA-damage by tobacco smoke. Carcinogenesis.

[16] Vahakangas KH, Bennett WP, Castren K, Welsh JA, Khan MA, Blomeke B, Alavanja MC, Harris CC (2001). p53 and K-ras mutations in lung cancers from former and never-smoking women. Cancer research.

[17] Nortier JL, Martinez MC, Schmeiser HH, Arlt VM, Bieler CA, Petein M, Depierreux MF, De Pauw L, Abramowicz D, Vereerstraeten P, Vanherweghem JL (2000). Urothelial carcinoma associated with the use of a Chinese herb (Aristolochia fangchi). The New England journal of medicine.

[18] Hsieh SC, Lin IH, Tseng WL, Lee CH, Wang JD (2008). Prescription profile of potentially aristolochic acid containing Chinese herbal products: an analysis of National Health Insurance data in Taiwan between 1997 and 2003. Chinese medicine.

[19] Olivier M, Eeles R, Hollstein M, Khan MA, Harris CC, Hainaut P (2002). The IARC TP53 database: new online mutation analysis and recommendations to users. Human mutation.

[20] Hinds PW, Finlay CA, Quartin RS, Baker SJ, Fearon ER, Vogelstein B, Levine AJ (1990). Mutant p53 DNA clones from human colon carcinomas cooperate with ras in transforming primary rat cells: a comparison of the “hot spot” mutant phenotypes. Cell growth & differentiation.

[21] Novara G, De Marco V, Dalpiaz O, Gottardo F, Bouygues V, Galfano A, Martignoni G, Patard JJ, Artibani W, Ficarra V (2008). Independent predictors of metachronous bladder transitional cell carcinoma (TCC) after nephroureterectomy for TCC of the upper urinary tract. BJU international.

[22] Zigeuner RE, Hutterer G, Chromecki T, Rehak P, Langner C (2006). Bladder tumour development after urothelial carcinoma of the upper urinary tract is related to primary tumour location. BJU international.

[23] Lai MN, Wang SM, Chen PC, Chen YY, Wang JD (2010). Population-based case-control study of Chinese herbal products containing aristolochic acid and urinary tract cancer risk. Journal of the National Cancer Institute.

[24] Lai MN, Lai JN, Chen PC, Tseng WL, Chen YY, Hwang JS, Wang JD (2009). Increased risks of chronic kidney disease associated with prescribed Chinese herbal products suspected to contain aristolochic acid. Nephrology.

[25] Tai YS, Chen CH, Huang CY, Tai HC, Wang SM, Pu YS (2015). Diabetes mellitus with poor glycemic control increases bladder cancer recurrence risk in patients with upper urinary tract urothelial carcinoma. Diabetes/metabolism research and reviews.

[26] Stapleton A (2002). Urinary tract infections in patients with diabetes. The American journal of medicine.

[27] Weldon TE, Soloway MS (1975). Susceptibility of urothelium to neoplastic cellular implantation. Urology.

[28] Kawai K, Kawamata H, Kemeyama S, Rademaker A, Oyasu R (1994). Persistence of carcinogen-altered cell population in rat urothelium which can be promoted to tumors by chronic inflammatory stimulus. Cancer research.

[29] Kantor AF, Hartge P, Hoover RN, Narayana AS, Sullivan JW, Fraumeni JF (1984). Urinary tract infection and risk of bladder cancer. American journal of epidemiology.

[30] Dunn SE, Kari FW, French J, Leininger JR, Travlos G, Wilson R, Barrett JC (1997). Dietary restriction reduces insulin-like growth factor I levels, which modulates apoptosis, cell proliferation, and tumor progression in p53-deficient mice. Cancer research.

[31] Zhao H, Grossman HB, Spitz MR, Lerner SP, Zhang K, Wu X (2003). Plasma levels of insulin-like growth factor-1 and binding protein-3, and their association with bladder cancer risk. The journal of urology.

[32] Baker L, Quinlan PR, Patten N, Ashfield A, Birse-Stewart-Bell LJ, McCowan C, Bourdon JC, Purdie CA, Jordan LB, Dewar JA, Wu L, Thompson AM (2010). p53 mutation, deprivation and poor prognosis in primary breast cancer. British journal of cancer.

[33] Chiaretti S, Tavolaro S, Marinelli M, Messina M, Del Giudice I, Mauro FR, Santangelo S, Piciocchi A, Peragine N, Truong S, Patten N, Ghia EM, Torrente I (2011). Evaluation of TP53 mutations with the AmpliChip p53 research test in chronic lymphocytic leukemia: correlation with clinical outcome and gene expression profiling. Genes, chromosomes & cancer.

[34] Levey AS, Bosch JP, Lewis JB, Greene T, Rogers N, Roth D (1999). A more accurate method to estimate glomerular filtration rate from serum creatinine: a new prediction equation. Modification of Diet in Renal Disease Study Group. Annals of internal medicine.

