# Supplementary material for: Recurrence pattern and TP53 mutation in upper urinary tract urothelial carcinoma
Source: Oncotarget. 2016 Jun 7;7(29):45225–36. doi: 10.18632/oncotarget.9904 (PMC5216718; doi:10.18632/oncotarget.9904)
Supplement: Supplementary file 1 [file oncotarget-07-45225-s001.pdf]

Recurrence pattern and TP53 mutation in upper urinary tract urothelial carcinoma

Supplementary Materials

Supplementary Table S1: Association between *TP53* mutation pattern and smoking status

TP53 mutation pattern	Smoking status	
	Smoking	No smoking
Mutations with A > T	6 (17%)	49 (38%)
Mutations other than A > T	11 (31%)	31 (24%)
No mutation	19 (53%)	49 (38%)

Supplementary Table S2: Association between *TP53* mutation pattern and bladder cancer history

TP53 mutation pattern	Bladder cancer history/recurrence		
	Previous/synchronous BC	No previous/synchronous BC	
		Recurrence after NU	No recurrence after NU
Mutations with A > T	16 (36%)	21 (31%)	27 (36%)
Mutations other than A > T	9 (20%)	16 (41%)	15 (20%)
No mutation	19 (43%)	11 (28%)	34 (45%)

BC = bladder cancer; NU = nephroureterectomy.
